# Physiological Responses and Partisan Bias: Beyond Self-Reported Measures of Party Identification

**DOI:** 10.1371/journal.pone.0126922

**Published:** 2015-05-26

**Authors:** Michael Bang Petersen, Ann Giessing, Jesper Nielsen

**Affiliations:** Department of Political Science, Aarhus University, Aarhus, Denmark; Defence Science & Technology Organisation, AUSTRALIA

## Abstract

People are biased partisans: they tend to agree with policies from political parties they identify with, independent of policy content. Here, we investigate how physiological reactions to political parties shape bias. Using changes in galvanic skin conductance responses to the visual presentation of party logos, we obtained an implicit and physiological measure of the affective arousal associated with political parties. Subsequently, we exposed subjects to classical party cue experiments where the party sponsors of specific policies were experimentally varied. We found that partisan bias only obtains among those exhibiting a strong physiological reaction to the party source; being a self-reported party identifier is not sufficient on its own. This suggests that partisan bias is rooted in implicit, affective reactions.

## Introduction

A citizen’s own position on a political policy appears to be shaped by their favored political party’s position, independent of actual policy content. This is demonstrated in a number of experimental studies where individuals’ opinions about policy proposals are shown to change when they are provided with cues about the parties’ positions on policies [1–6]. If citizens identify with a party supporting a policy, they tend to agree with it; if not, they reject the policy. In other words, people are biased partisans.

It is commonly argued that partisan bias emerges from psychological mechanisms designed to manage social identification [1, 6–8]. Hence, there is increasing evidence suggesting that one's party identification—like other group identifications—forms early in life, is transmitted across generations, and endures over one’s lifespan and in the face of significant changes in the environment [9–11]. Moreover, recent evidence shows that people intuitively treat party affiliations as group affiliations: They use the same psychological mechanisms to form impressions of the party affiliations of others as they do to form impressions about other types of group identities [8]. In this perspective, partisan bias arises from attempts to defend a cherished group identity: It is distressing to disagree with a group to which one feels emotionally attached [12] and this cognitive dissonance creates motivation for toeing the party line [4].

In demonstrating the effect of partisan bias, however, studies have exclusively relied on self-reported measures of party identification. The preferred self-reported measure was devised in the 1950s on the basis of contemporary standards for attitude research and knowledge about survey responses [7]. From the perspective of cumulative research, there are many advantages to the consistent use of a specific measure across multiple decades. At the same time, knowledge about the measurement problems relating to an exclusive reliance on self-reported answers has grown exponentially in recent decades [13–15]. These problems are also evident in the context of self-reported measures of party identification [16–17]. While the party identification measure was designed to gauge “the individual’s *affective* orientation” towards a party (p. 121 in [7], our emphasis), recent research has demonstrated that the measure is conflated because it also taps a number of instrumental considerations [16–18]—in particular, the proximity between the voter’s general preferences and the party’s overall positions [19].

As consequence, extant research has left untested the key assumption that partisan bias emerges predominantly from an affective attachment to the party. This is unfortunate because a number of studies have argued that partisan bias could in fact emerge from non-affective, instrumental considerations [20–22]. Forming opinions on new political policies is complex. It is possible that partisan bias reflects an instrumentally adopted heuristic to minimize cognitive effort by simply opting for whatever position the preferred party takes.

In this article, we report findings based on a direct measure of the affect associated with political parties and demonstrate how partisan bias is specifically tied to the affective component of party identification. To accomplish this, we followed recent studies on political orientations and utilized measurement techniques originally developed in psychophysiology to measure activity in the sympathetic nervous system—a system responsible for the regulation of affective arousal—in order to measure the affective basis of subjects’ party attachments [23–24]. Specifically, we used a laboratory experiment to measure changes in galvanic skin conductance responses (SCR) to the visual presentation of party logos. As summarized in recent work (p. 10 in [25]), it is “well established that SCR covaries with the arousal dimension of affect, indexing its intensity” and, hence, the setup provided a measure of whether the associations primed by the party logo were affectively charged or not. Subsequently, subjects were exposed to classical party cue experiments where the party sponsor of specific policy proposals was experimentally varied, and we analyzed how self-reported party affiliation and the physiological reactions to party logos influenced the effect of these party cues. We found that the classic partisan bias only obtains among those who exhibited a strong physiological reaction to the party sponsor; being a self-reported party identifier was not sufficient on its own. This provides strong evidence that specifically the *affective* component of party identification drives partisan bias.

## Design and Methods

### Participants and Procedure

Subjects were recruited at the campus of a large Danish research university. As incentives, subjects participated in a lottery of six gift certificates with a value of $40 each. A total of 58 subjects participated in the experiment (27 males and 31 females; mean age = 23.6, range: 19–32). Participants were thoroughly briefed before the study and written consent was obtained from all. Denmark does not have an institutional review board for research outside biomedical research. In accordance with the national scientific guidelines, formal ethical approval for the present research was not obtained (see http://www.cvk.sum.dk/cvk/home/english.aspx).

Upon entering the laboratory, the subjects were given a short briefing, filled out consent forms and were asked to disinfect their hands. The subjects were then seated in front of a computer and had disposable electrodes (EL507) attached to the middle and index fingers of the non-dominant hand. These electrodes were connected to a BioPac MP150 with a GSR100C module, which is specifically built to obtain measures of skin conductance. Subsequently, the subjects completed a brief computer-administered survey on party attachment. After this survey, the collection of data on skin conductance responses was initiated. After reading a brief introduction on the screen, subjects were shown a slideshow of images in randomized order while their skin conductance reactions were measured. Here, the subjects did not answer any questions and no significant cognitive effort was required of the subjects.

**Fig 1:**
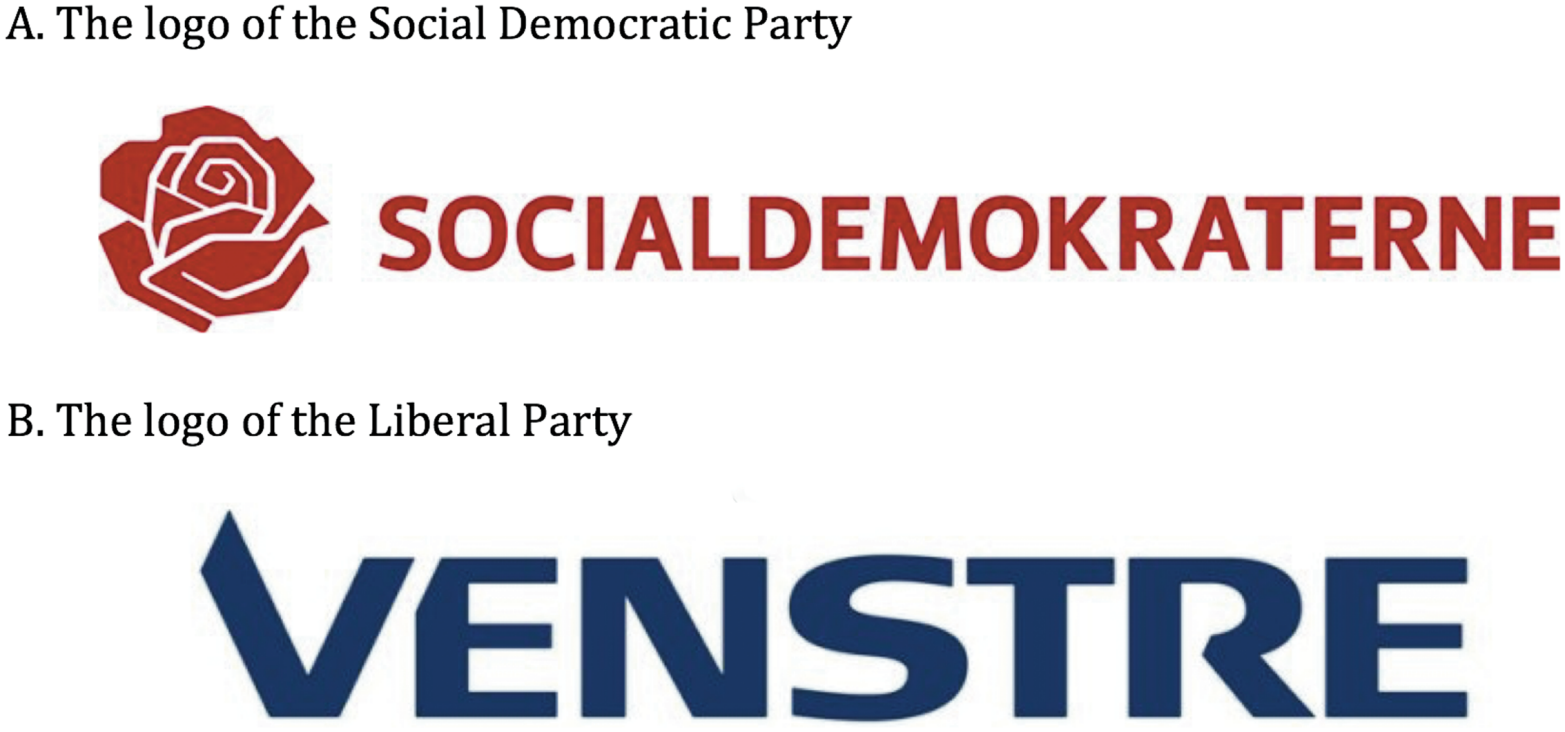
The party logos utilized to elicit skin conductance responses and to indicate party provenance of the policy proposals.

Each image was visible for ten seconds, followed by a blank white screen for another ten seconds (as recommended by [25]). The interstimulus interval was necessary to ensure that the skin conductance reactions had returned to their baseline level before the next image was shown. Logos of the two largest Danish political parties, The Liberal Party and The Social Democrats, were included (see Fig 1). Currently, eight Danish parties hold seats in Parliament. Using the two major parties maximized the likelihood that a substantial proportion of the subjects were affiliated with one of the parties. Furthermore, these two parties ensured that both a center-left and a center-right party were tested. In addition to the party logos, some non-political images were included. These were highly emotional images, such as a spider, an infected foot, and a happy child, as well as neutral images, such as a light bulb, an unknown man, and an unknown woman.

After completion of the skin conductance measurements, the subjects were exposed to a traditional party cue experiment. Here the party logos from the skin conductance measurements were randomly assigned to 16 policy proposals. The given logo assigned to a political proposal appeared on the screen above the proposal and subjects were informed that the logo for a given proposal indicated provenance (see S1 Fig for an example). Because the nature of the policy issues could affect the observed party cue effect [6], we included proposals concerning a wide range of policy issues, economic as well as non-economic. An example of the former proposal was that income tax should be reduced, and of the latter that police officers should not be allowed to wear religious symbols.

A full list of proposals is provided in S1 Table. In addition to the 16 policy proposals attributed to a political party, the subjects responded to eight policy proposals that were not attributed to a specific party. These additional proposals served as placebo tests and are analyzed in the S2 Table. To the extent that the effects we report in the main text are driven by partisan bias, they should not obtain when no party sponsor is present.

After completing the experiment, the subjects were given a thorough debriefing.

### Measures

Our setup allows us to measure three key variables: traditional explicit measures of party attachment, an implicit measure of party affect and, as the dependent variable, degree of agreement with proposals advocated by specific political parties. Furthermore, we obtained a number of control variables.

### Self-Reported Measures of Party Affiliation

We obtained two traditional measures of party affiliation. First, we obtained the traditional measure of party identification applied to the Danish context (as used in the Danish National Election Survey). While the classical measure was devised for an American context, research shows that it is highly predictive of vote choice in a Danish context [26]. We created a separate identification measure for each party that reflected whether the subjects identified with the party (a value of 1.0), were leaning towards the party (a value of .5) or neither identified with nor leaned towards the specific party (a value of 0). In S2 Fig, the distribution of party identification in the sample is displayed.

Second, we obtained ratings of sympathy toward each party (“Do you like or dislike the following parties?”) on an 11-point scale (1 = “Really dislike the party” and 11 = “Really like the party”). Some prior research suggests that such “thermometer ratings” of sympathy are more reliable indicators of party attachments in multiparty systems such as the Danish one [27]. Because of the abundance of parties, only a segment of subjects will explicitly label themselves as identifiers with one of the two parties. In this respect, sympathy scores are preferable because they allow us to gauge fine-grained differences in levels of attachment between all subjects rather than simply lump most of our subjects into one large “non-identifier” category. In S3 Fig, distributions of party sympathies in the sample are shown. Following previous research on party sponsor effects [3], we use collapsed versions of these two party attachment measures in the analyses. Specifically, we created two measures, termed Identification with Party and Sympathy for Party, tapping the attachment to the party sponsoring the proposal in the specific experimental condition. Thus, in the conditions in which the Liberal Party proposed a policy, these measures reflected attachment to the Liberal Party and, conversely, attachment to the Social Democrats in the conditions in which they were identified as having proposed a policy.

#### Measure of Physiological Response to Party

SCR implicitly indexes activity in the sympathetic nervous system, which is crucially involved in affective processes [28]. Importantly, SCR measures the strength of emotions, not whether the affection is positive or negative. By gauging SCR to the logos of the Liberal Party and the Social Democrats, we are in effect provided with an implicit, physiological measure of the affective arousal associated with these parties. As described above, SCR was obtained using the BioPac system. On the GSR100C module, a low pass filter of 1 Hz was set and the gain switch was set to 5 μmho/V.

Information from the BioPac was recorded through AcqKnowledge 4.0. Here a sampling rate of 1000 Hz was utilized for data acquisition and the data was filtered using a software-based high pass filter of 0.05 Hz. AcqKnowledge was also utilized to record the timing of the stimuli presentation. Specifically, the visual stimuli were presented on a computer screen using E-Prime 2.0. The presentation computer was attached to the BioPac MP150 unit. When an image was presented and terminated, E-Prime sent a signal to the unit, which was recorded by Acknowledge. The simultaneous recording of the SCR signal and the presentation signal allowed us to determine the precise physiological reaction to an image with high levels of precision.

To quantify the SCR, we followed recent recommendations (p. 167 in [25]) and used the area bounded by a curve, which “captures both the amplitude and the temporal characteristics of an SCR, and therefore is likely to be a more valid indicator than either aspect alone.” The window of interest that delineates the area starts one second after the stimulus is presented and ends ten seconds later. This was done to minimize the risk of recording non-event-specific SCRs. To ensure symmetrically and approximately normally distributed data, a logarithmic transformation was applied to the SCR variables. As with the explicit party attachment measures, the final measure reflects SCR towards the specific party in each experimental condition.

#### Agreement with Party

As the dependent variable, for each policy proposal in the party cue experiment, we asked subjects how strongly they agreed or disagreed with the proposal. Seven-point Likert scales were used.

#### Control Variables

Because it is plausible that some individuals will display strong SCRs for extraneous reasons (e.g. due to thickness of skin), it is necessary to establish an individual SCR baseline (see also [23]). This was obtained by controlling for the subjects’ general reactions towards stimuli in the analyses below. The general reaction was measured as the subjects’ reactions towards the presented non-political pictures (e.g. a flower and a spider). The reactions were composed into a scale (α = .88). In total, reactions towards eight non-political pictures were obtained. S3 Table provides the full list.

In addition to the SCR baseline measure, several self-reported control variables were obtained. We measured subjects’ sex, their age, their highest completed level of education (elementary school, high school, some college or college degree) and their political ideology measured as self-reported placement on an 11-point scale with the endpoints labeled “Left” (0) and “Right” (10).

### Analyses

All variables have been recoded to vary between 0 and 1, except age which is measured in years. Analyses were performed in Stata 13 and, due to the interval-scaled nature of the dependent variable, we utilized multivariate OLS regression analyses to estimate the effect of party attachments on agreement with a party’s policy proposals. It could be argued that the dependent variable—measured on a 7-point scale—is ordinal-scaled. In such a case, an ordered probit model would be more appropriate for the data. To examine the robustness of our conclusions, we therefore repeated the key analyses using ordered probit models.

Tests of the crucial assumptions of OLS regression models—linear relationship between the dependent and independent variable, lack of outliers, normally distributed error terms, homoscedasticity and lack of multicollinearity—are presented for the key models (Models 3 and 4 in Table 1) in S4 Table and S4 Table. In general, these tests show that the OLS regression model is applicable for the present data. The assumption of homoscedasticity is violated but was corrected for using robust standard errors. Also, the models show signs of multicollinearity. This, however, is expected in any regression model with interaction terms and leaves the coefficients unbiased [29].

**Table 1:** Effects of cognitive and physiological measures of party attachment on agreement with party.

Model	1	2	3	4
Female	-0.01 (0.02)	-0.01 (0.02)	-0.01 (0.02)	-0.02 (0.02)
Age	0.01** (0.00)	0.01* (0.00)	0.01* (0.00)	0.01* (0.00)
Education	-0.00 (0.06)	0.01 (0.05)	0.01 (0.06)	-0.01 (0.06)
Political Ideology	0.08 (0.04)	0.07 (0.04)	0.08 (0.04)	0.06 (0.04)
Baseline SCR	-0.06 (0.07)	-0.04 (0.08)	-0.08 (0.07)	-0.03 (0.08)
SCR towards Party	0.05 (0.07)	0.03 (0.07)	0.02 (0.07)	-0.15 (0.09)
Identification with Party	0.13** (0.04)	-	-0.10 (0.09)	-
Sympathy towards Party	-	0.13*** (0.04)	-	-0.04 (0.08)
SCR towards Party * Identification	-	-	0.47* (0.20)	-
SCR towards Party * Sympathy	-	-	-	0.37* (0.16)
Constant	0.16 (0.10)	0.14 (0.10)	0.20* (0.10)	0.25* (0.11)
Observations	894	894	894	894
*R* ^*2*^	0.217	0.218	0.221	0.222

Notes: Entries are unstandardized OLS regression coefficients with standard errors in parentheses. Cluster robust standard errors with subject ID as cluster variable are used. All models include dummy variables for each individual policy (these are not displayed in the table). All variables are scaled 0–1 except age, which is measured in years.

*p < .05,

**p < .01,

***p < .001,

two-tailed p-values reported.

As effect size measures, we report unstandardized regression coefficients, which—given the 0–1 coding of the variables—can be interpreted as the change in percentage points of the full dependent scale when the independent measure changes from its lowest to its highest value [30].

Our unit of analysis is expressions of agreement with a specific policy proposal. Specifically, the 58 subjects expressed their level of agreement with 16 proposals, providing us with 58 × 16 = 928 observations for analyses. To correct for the autocorrelation emerging from the fact that multiple observations are produced by a single subject, we utilize cluster robust standard errors with subject ID as the cluster variable. Because a small number of subjects failed to provide information on one or more measures, we end up with a complete sample of 894 observations.

All analyses were controlled for the baseline SCR measure as well as the sex, age, education and political ideology of the subject. In addition, we controlled for the individual policy proposals in the form of a series of dummy variables. In this way, all potential differences between the proposals have been controlled for.

Replication data and files for statistical analyses are available at the public data repository Dataverse (www.thedata.org): http://dx.doi.org/10.7910/DVN/GJDMSV.

## Results

As a starting point, we analyzed the extent to which normal partisan bias was obtained in our study; that is, whether subjects tended to agree with the party with which they expressed an affiliation. These analyses are represented in Table 1, Models 1 and 2. Here, we regress agreement with the policy proposals on the two self-reported measures of party affiliation. For both measures, we find highly significant and positive effects of affiliation on agreement. Consistent with previous studies, subjects agree more when policy proposals are attributed to a party with which they are affiliated.

To investigate the predicted affective underpinnings of this effect, Models 3 and 4 include two-way interaction terms between self-reported measures of party affiliation and physiological arousal associated with the party in the form of changes in skin conductance in response to the party logo. This allows us to investigate whether partisan bias is present independently of affective arousal or whether the bias only obtains among those whose party affiliation is associated with affect. We find significant interaction effects for both affiliation measures. Hence, the strength of physiological responses to the party moderates the traditional party cue effect of expressed identification or sympathy. As a test of robustness, we also tested the predicted interaction effects using ordered probit models, which do not assume that the dependent variable is interval-scaled. In these models the interactions reported in both Model 3 (χ^2^(1) = 5.84, p = .0016) and Model 4 (χ^2^(1) = 5.44, p = 0.020) continue to be significant.

**Fig 2:**
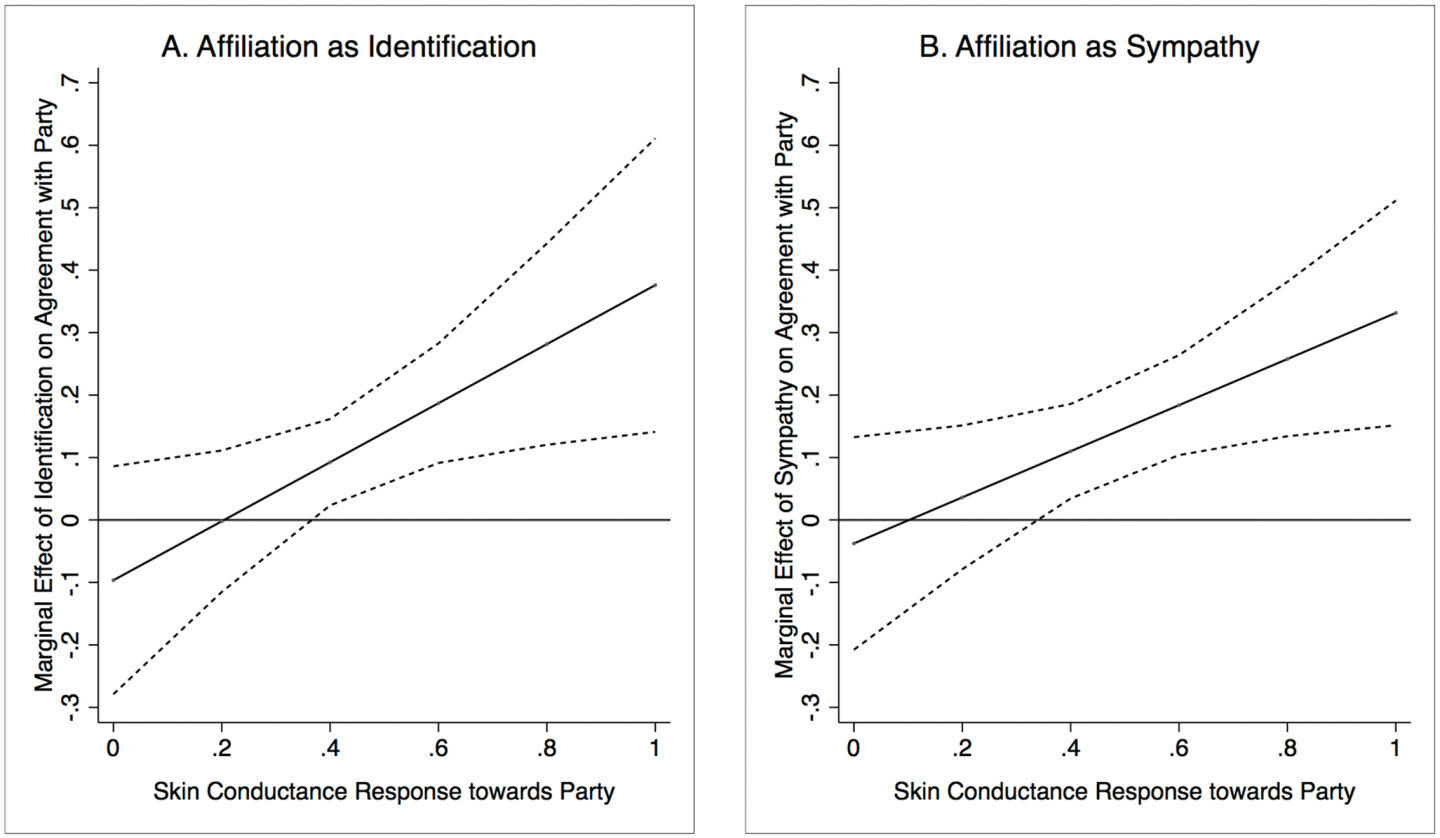
Predicted marginal effects of affiliation with party on agreement with party with associated confidence intervals. Notes. Marginal effect plots with 95% confidence intervals are computed on the basis of Table 1.

To interpret this effect, Fig 2 displays the predicted marginal effects of the affiliation measures (with 95% confidence intervals) on agreement. Two important observations are immediately apparent from Fig 2: First, there is a positive effect of physiological responses such that traditional party affiliation measures become more and more predictive of agreement as physiological responses to the party increase. In other words, individuals who are physiologically aroused by the party are highly susceptible to be biased partisans. In terms of effect size, this effect is relatively strong: among those who are most physiologically aroused, the effect of affiliation on agreement is between .30 and .40 (depending on the specific measure). This implies that those are most affiliated with a party are .30–.40 percentage points more in agreement with the party than those who are unaffiliated. Second, for both affiliation measures, there are no effects of self-reported party affiliation for those who do not also experience a physiological response to the party. That is, if the party does not spark an affective response, we find that individuals neither agree nor disagree with the party as a function of party identification or party sympathy. Perceiving oneself as a party supporter does not make an individual more inclined to follow a party on specific policy proposals unless this affiliation is steeped in physiologically real affect.

As described in above, subjects also responded to eight policy proposals that were not attributed to a specific party. To the extent that the effects we report above are driven by partisan bias, they should not obtain when no party sponsor is present. This is analyzed in S2 Table. Consistent with the partisan bias-oriented interpretation of the above findings, the results in S2 Table show that party affiliation (whether measured using self-reports or SCR) does not enhance agreement with proposals when these proposals are not attributed to a specific party. This provides strong additional support for the claim that the effects of our physiological measure do indeed reflect a measure of affective affiliation with the party sponsor.

## Conclusions

These findings extend extant research in two important ways. First of all, they provide additional evidence of the need to distinguish between the instrumental and affective components of party identification [16–17, 31]. Hence, we find distinct effects among party identifiers who do not show signs of affective reactions to their party relative to those identifiers who do display strong affective reactions. Given that the implicit, physiological measure of affect is not filtered through instrumental considerations, as all self-reported measures necessarily are, this measure provides a much more reliable and valid measurement of affective processes [13–15]. Hence, using this improved measure, we have been able to solidify the conclusion that the original measure of party identification does not always tap the deep affective orientations it was designed to [7].

Second, based on this physiological measure of party affect, we have demonstrated that affect drives partisan bias, i.e., the tendency for party affiliates to toe the party line on policies and issues. A purely instrumental affiliation with a party does not give rise to bias unless paired with a strong physiological response to the party. This suggests that partisan bias stems from motivated attempts to defend affectively grounded group attachments [4, 8].

These contributions notwithstanding, it is important to note the limitations of the present study. First, the findings of this study should not be taken as an indication that partisan bias dominates opinion formation in politics. The substance of policies and debates certainly matters and, as argued in recent work [32], perhaps more so than partisanship. This is also reflected in the fact that in itself the partisan affiliation measures only explain around 2–3% of the variation in the data, despite the substantial effect size. The bulk of the explained variation comes from the dummies measuring which specific policy proposal the subject has responded to. Nonetheless, partisan bias is also a factor, and the finding of the present study is that it emerges from affective processes. Second, the utilized sample is a narrow convenience sample. For reasons of external validity, it will be important for future research to seek to replicate these findings on more representative samples of general populations. Such a sampling strategy will constitute an obstacle to measuring party affect using psychophysiological techniques in the laboratory, but alternative measurement techniques (e.g., the implicit association test [33]) could be administered online to broader samples. Third, and also related to external validity, the findings of this study are based on observations from a single country: Denmark. Denmark is a multi-party system with a proportional election system and high levels of political participation. One question is whether the findings are replicable in countries that do not share these characteristics. One country that is different from Denmark on multiple political dimensions is the United States. However, if anything, other studies suggest that the effects of partisan affect might be even stronger in highly polarized and antagonistic two-party systems such as the American [34]. How such contextual factors regulate the effects of partisan affect is another key question for future research.

## Supporting Information

S1 Table List of policy proposals. (DOCX)

S2 Table Effects of cognitive and physiological measures of party attachment on agreement with policy statements without specific party sponsor. (DOCX)

S3 Table List of non-political images used to obtain the baseline SCR measure. (DOCX)

S4 Table Tests of OLS regression assumptions for model with Sympathy towards Party as affiliation measure. (DOCX)

S5 Table Tests of OLS regression assumptions for model with Identification with Party as affiliation measure. (DOCX)

S1 Fig Sample stimuli used in the party cue experiment. (DOCX)

S2 Fig Sample distribution of party identification. (DOCX)

S3 Fig Sample distribution of sympathy towards the Social Democratic Party and the Liberal Party. (DOCX)
