# Supplementary material for: Physiological Responses and Partisan Bias: Beyond Self-Reported Measures of Party Identification
Source: PLoS One. 2015 May 26;10(5):e0126922. doi: 10.1371/journal.pone.0126922 (PMC4444316; doi:10.1371/journal.pone.0126922)
Supplement: S1 Table — (DOCX) [file pone.0126922.s001.docx]

**S1 Table. List of policy proposals.**

| 1. Everyone applying for a residence permit should be required to receive education on the Danish language and culture. |
| --- |
| 1. The use of genetically modified fodder should be banned. |
| 1. Child allowance should only be provided to those with greatest need. |
| 1. Denmark should support the development of a missile shield with bases in Europe. |
| 1. Denmark should join the Euro right away. |
| 1. Denmark should be reluctant to provide military contributions to dangerous international missions. |
| 1. The environmental taxes on the most polluting cars should be increased. |
| 1. We should launch a tax reform that makes it more attractive to work. |
| 1. We should introduce a mandatory minimum punishment of two years in jail for rape. |
| 1. We should reform the early retirement scheme such that fewer dpeople can use it. |
| 1. The EU should not admit countries with non-Western values. |
| 1. To attract foreign labour we should maintain a low corporation tax. |
| 1. It should be possible for homosexual couples to get married in church. |
| 1. Solitary confinement should be used less than is the case today. |
| 1. We should not lower the social welfare benefits to pressure the unemployed to apply for jobs. |
| 1. The police should not wear religious symbols. |
| 1. The police should be far more visible in the street life. |
| 1. The police should get better possibilities for using surveillance in their investigations. |
| 1. We should give immigrants a lower hourly wage than Danes in order to get immigrations employed. |
| 1. We should abolish the law against blasphemy that bans degrading statements about people's religion. |
| 1. We should lower the tax on income. |
| 1. We should by law ensure that there is a maximum of 22 pupils in each elementary school class. |
| 1. We should increase the use of surveillance cameras in public places. |
| 1. Spouses to Danes below age 24 should be allowed to get a residence permit in Denmark. |

Notes. This is a translated list of the proposals used in the study. The list of the proposals is a mix of newly formulated proposals and proposals from Petersen, M.B., Skov, M., Serritzlew, S. & Ramsöy, T. (2013), “Political Parties and Motivated Reasoning: Evidence for Increased Processing in the Face of Party Cues”, *Political Behavior*, *35*(4), 831-854. In the present study, the order of the proposals was randomized. 8 of the proposals were randomly attributed to the Social Democratic Party, 8 of the proposals were randomly attributed to the Liberal Party and 8 of the proposals were attributed to no particular party ("A party"). We analyze agreement with the latter proposals in Supplementary Information S1.
