# Supplementary material for: Physiological Responses and Partisan Bias: Beyond Self-Reported Measures of Party Identification
Source: PLoS One. 2015 May 26;10(5):e0126922. doi: 10.1371/journal.pone.0126922 (PMC4444316; doi:10.1371/journal.pone.0126922)
Supplement: S2 Table — (DOCX) [file pone.0126922.s002.docx]

**S2 Table. Effects of cognitive and physiological measures of party attachment on agreement with policy statements without specific party sponsor.**

| Model | 1 | 2 | 3 | 4 |
| --- | --- | --- | --- | --- |
| Female | -0.01  (0.03) | -0.00  (0.04) | -0.00  (0.04) | 0.01  (0.03) |
| Age | 0.00  (0.01) | 0.00  (0.00) | 0.00  (0.01) | 0.01  (0.00) |
| Education | -0.03  (0.10) | -0.01  (0.09) | -0.02  (0.10) | -0.02  (0.10) |
| Political Ideology | 0.09  (0.08) | 0.06  (0.10) | 0.09  (0.08) | 0.10  (0.11) |
| Baseline SCR | 0.08  (0.13) | 0.12  (0.13) | 0.09  (0.12) | 0.12  (0.12) |
| SCR towards Liberal Party | -0.02  (0.11) | -0.05  (0.10) | -0.03  (0.10) | 0.22  (0.13) |
| SCR towards Social Democratic Party | 0.01  (0.08) | -0.01  (0.07) | 0.01  (0.07) | 0.06  (0.15) |
| Identification with Social Democratic Party | 0.04  (0.05) | - | 0.06  (0.20) | - |
| Identification with Liberal Party | 0.05  (0.08) | - | 0.02  (0.12) | - |
| Sympathy towards Social Democratic Party | - | 0.01  (0.08) | - | 0.04  (0.14) |
| Sympathy towards Liberal Party | - | 0.07  (0.08) | - | 0.31^**^  (0.09) |
| SCR * Identification (Liberal Party) | - | - | 0.07  (0.27) | - |
| SCR * Identification (Social Democratic Party) | - | - | -0.05  (0.36) | - |
| SCR * Sympathy (Liberal Party) | - | - | - | -0.55^***^  (0.15) |
| SCR * Sympathy (Liberal Party) | - | - | - | -0.10  (0.26) |
| Constant | 0.24  (0.17) | 0.23  (0.16) | 0.25  (0.19) | -0.03  (0.17) |
| Observations | 448 | 448 | 448 | 448 |
| *R*^2^ | 0.237 | 0.235 | 0.237 | 0.246 |

Notes. Entries are unstandardized OLS regression coefficients with standard errors in parentheses. Cluster robust standard errors with subject ID as cluster variable are used. All models include dummy variables for each individual policy (these are not displayed in the table). All variables are scaled 0-1 except age, which is measured in years. ^*^p <.05, ^**^p<.01, ^***^p<.001, two-tailed p-values reported. Consistent with the partisan bias-oriented interpretation of the effects reported in the main text, the results show that party affiliation (whether measured using self-reports or SCR) does not enhance agreement with proposals, when these proposals are not attributed to a specific party. In Model 4, we observe a single significant interaction term but this interaction is in the *opposite* direction than those observed in the main text. For unknown reasons, when there is no party sponsor, the self-reported and physiological measures of affiliation with the Liberal Party work in opposite directions (although, this is not replicable for affiliation with the Social Democratic Party).
