# Supplementary material for: Physiological Responses and Partisan Bias: Beyond Self-Reported Measures of Party Identification
Source: PLoS One. 2015 May 26;10(5):e0126922. doi: 10.1371/journal.pone.0126922 (PMC4444316; doi:10.1371/journal.pone.0126922)
Supplement: S3 Table — (DOCX) [file pone.0126922.s003.docx]

**S3 Table. List of non-political images used to obtain the baseline SCR measure.**

| 1. Cherry flowers on a white background |
| --- |
| 1. A happy baby playing with three yellow rubber ducks against a white background |
| 1. A foot with a large infected wound against a background of grass |
| 1. A large black spider on a white background |
| 1. The socket of a light bulb against a black background |
| 1. An adjustable wrench against a wooden background |
| 1. A head shot of a well-dressed middle-aged man against a grey background |
| 1. A head shot of a well-dressed middle-aged woman against a grey background |
