# Supplementary material for: Physiological Responses and Partisan Bias: Beyond Self-Reported Measures of Party Identification
Source: PLoS One. 2015 May 26;10(5):e0126922. doi: 10.1371/journal.pone.0126922 (PMC4444316; doi:10.1371/journal.pone.0126922)
Supplement: S4 Table — (DOCX) [file pone.0126922.s004.docx]

**S4 Table. Tests of OLS regression assumptions for model with Sympathy towards Party as affiliation measure.**

| **Assumption** | **Test** |
| --- | --- |
| Linearity | Augmented Component-Plus-Residual Plot with Lowess curve:    The plot does not show any strong deviation from linearity for the key variable of interest: the interaction between SCR towards Party × Sympathy towards Party. |
| Lack of influential observations | 38 observations with a Cook's Distance above 4/e(N).  Test of interaction term without inclusion of these influential observations: F_1,55_=8.75, p=0.0045.  This test demonstrates that the predicted interaction effect is not caused by outliers. |
| Normally distributed error terms | Q-Q-Plot:    The plot does not show any strong deviation from normality. |
| Homo-scedasticity | White's test for Ho: homoscedasticity: **χ**^2^_249=_ 326.65, p < 0.01  (performed on model without cluster robust standard errors)  The test provides evidence of heteroscedasticity which is corrected for using cluster robust standard errors. |
| Lack of multi-collinearity | Variables with a VIF below .30: Sympathy towards Party, SCR towards Party, Sympathy towards Party ×SCR towards Party. These are the interaction variables, which is expected and leaves the estimates unbiased. |

Notes. Tests based on recommendations in Sønderskov, K. M. (2015). *Stata: A Practical Introduction*. Copenhagen: Hans Reitzel Press.
