# Supplementary material for: Physiological Responses and Partisan Bias: Beyond Self-Reported Measures of Party Identification
Source: PLoS One. 2015 May 26;10(5):e0126922. doi: 10.1371/journal.pone.0126922 (PMC4444316; doi:10.1371/journal.pone.0126922)
Supplement: S1 Fig — (DOCX) [file pone.0126922.s006.docx]

**S1 Fig. Sample stimuli used in the party cue experiment.**

**
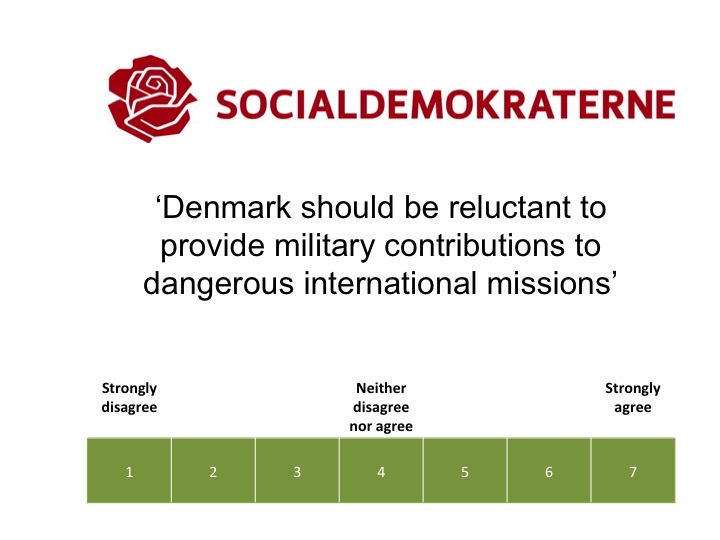
**

Notes. This is a translated example of the stimuli used in the experiment. This displays a condition where the proposal 6 (see Table A1) is attributed to the Social Democratic Party by use of the party logo. In the other conditions for this proposal, the logos of the Social Democratic Party were replaced with the logo of the Liberal Party (see Figure 1 in the main text) or, for the placebo tests, simply the sentence "A party" (presented in black font).
