# Supplementary material for: Physiological Responses and Partisan Bias: Beyond Self-Reported Measures of Party Identification
Source: PLoS One. 2015 May 26;10(5):e0126922. doi: 10.1371/journal.pone.0126922 (PMC4444316; doi:10.1371/journal.pone.0126922)
Supplement: S2 Fig — (DOCX) [file pone.0126922.s007.docx]

**S2 Fig. Sample distribution of party identification.**

Notes. N = 58. The figure shows the distribution of party identification in the sample. The 'Identification with Party' variable used in the main text has a value of 1 for Social Democratic Party Identifiers and a value of .5 for Social Democratic Party Leaners when the party sponsor is the Social Democratic Party and a value of 1 for Liberal Party Identifiers and a value of .5 for Liberal Party Leaners when the party sponsor is the Liberal Party. For all others, the value of the measure is 0. In the analyses used in Table A5, two measures of identification were formed: one for the Social Democratic Party (1, .5 and 0 for identifiers, leaners and neither, respectively) and one for the Liberal Party. The values of these were 1, .5 and 0 for identifiers, leaners and neither, respectively.
