# Supplementary material for: Physiological Responses and Partisan Bias: Beyond Self-Reported Measures of Party Identification
Source: PLoS One. 2015 May 26;10(5):e0126922. doi: 10.1371/journal.pone.0126922 (PMC4444316; doi:10.1371/journal.pone.0126922)
Supplement: S3 Fig — (DOCX) [file pone.0126922.s008.docx]

S3 Fig. Sample distribution of sympathy towards the Social Democratic Party and the Liberal Party.

Notes. N = 58. The figure shows the distribution of party sympathies in the sample. A higher value indicates higher sympathy. The 'Sympathy towards Party' variable used in the main text equals the score of the sympathy measure for the party sponsoring the proposal in question. In the analyses used in Table A5, the sympathies for the two parties are entered as separate variables.
